# An Integrated Metabolomic Study of Osteoporosis: Discovery and Quantification of Hyocholic Acids as Candidate Markers

**DOI:** 10.3389/fphar.2021.725341

**Published:** 2021-08-06

**Authors:** Dawei Deng, Chen Pan, Zeming Wu, Yujiao Sun, Chang Liu, Hong Xiang, Peiyuan Yin, Dong Shang

**Affiliations:** ^1^ Department of General Surgery, First Affiliated Hospital of Dalian Medical University, Dalian, China; ^2^ Clinical Laboratory of Integrative Medicine, First Affiliated Hospital of Dalian Medical University, Dalian, China; ^3^ Department of Hepato-biliary-pancreas, Affiliated Hospital of North Sichuan Medical College, Nanchong, China; ^4^ iPhenome biotechnology (Yun Pu Kang) Inc, Dalian, China; ^5^ Institute of Integrative Medicine, Dalian Medical University, Dalian, China

**Keywords:** osteoporosis, lipids, bile acids, ageing, metabolomics

## Abstract

Osteoporosis is becoming a highly prevalent disease in a large proportion of the global aged population. Serum metabolite markers may be important for the treatment and early prevention of osteoporosis. Serum samples from 32 osteoporosis and 32 controls were analyzed by untargeted metabolomics and lipidomic approaches performed on an ultra-high performance liquid chromatography and high-resolution mass spectrometry (UHPLC-HRMS) system. To find systemic disturbance of osteoporosis, weighted gene correlation network analysis (WGCNA) and statistical methods were employed for data-mining. Then, an in-depth targeted method was utilized to determine potential markers from the family of key metabolites. As a result, 1,241 metabolites were identified from untargeted methods and WGCNA indicated that lipids metabolism is deregulated and glycerol phospholipids, sphingolipids, fatty acids, and bile acids (BA) are majorly affected. As key metabolites of lipids metabolism, 66 bile acids were scanned and 49 compounds were quantified by a targeted method. Interestingly, hyocholic acids (HCA) were found to play essential roles during the occurrence of osteoporosis and may be potential markers. These metabolites may be new therapeutic or diagnosis targets for the screening or treatment of osteoporosis. Quantified measurement of potential markers also enables the establishment of diagnostic models for the following translational research in the clinic.

## Introduction

Osteoporosis is a progressive systemic bone disease that is characterized by bone loss and microstructural deterioration and results in increased bone fragility, which affects over 200 million people worldwide (Curtis et al., 2017; Compston et al., 2019). Complications of osteoporosis such as chronic pain, fracture and disability seriously affect the quality of life of elderly individuals. Fracture is the most serious complication, with more than 8.9 million osteoporosis-related fractures occurring annually (Cruz-Jentoft and Sayer, 2019). As the global population ages, osteoporosis and its complications are becoming an increasingly serious public health burden (Sànchez-Riera et al., 2014; Tarrant and Balogh, 2020). Osteoporosis is also a highly insidious disease. Due to the absence of obvious symptoms and sensitive biomarkers, many patients are diagnosed only after a fracture has occurred (Wang et al., 2019; Fang et al., 2020). Furthermore, the first-line drugs used to treat osteoporosis are associated with a substantial number of complications, and the overall therapeutic effects are unsatisfactory (Park et al., 2013; Komm et al., 2015). This indicates that we do not fully understand osteoporosis and the therapeutic targets required.

Metabolites are the ultimate functional products that manifest both genetic and environmental variations, and they combine external stimuli with intracellular signals (Peng et al., 2015). Metabolic profiles obtained using metabolomics techniques under different conditions are closely related to human health (Leslie and Beyan, 2011). Osteoporosis is a metabolic bone disease, and studies have indicated that significant changes in endogenous metabolites modulate bone remodelling in a mouse model of osteoporosis (Nam et al., 2018). Moreover, in the treatment of osteoporosis, oestradiol changed 27 intracellular metabolite levels by correcting lipid and amino acid disorders (Liu et al., 2015). Metabolomics characterizes metabolites in biological samples to provide information on pathway activity, which provides a suitable approach for the study of osteoporosis. Non-targeted metabolomics platforms aim to enlarge the coverage of endogenous metabolites for a better understanding metabolic pathways or screening potential biomarkers. Thus, the challenges for untargeted metabolomics are detection, discovery and identification of differential metabolites. Targeted metabolomics methods focused on a limited number of compounds and provides sensitive and precise measurement of metabolites. The combination of the two approaches has greatly facilitated the discovery of biomarkers and the understanding of pathophysiological mechanisms (Xuan et al., 2020).

Changes in human serum metabolites might reflect pathophysiological alterations caused by various diseases (Shu et al., 2020, 19). Here, metabolic alternations in patients with osteoporosis were analyzed by untargeted metabolomic and lipidomic methods. To better understand the metabolic deregulations occurred in the patients, WGCNA algorithm and multivariate statistical methods were applied. Then targeted metabolomics method performed on a triple quadrupole MS was employed to obtain an in-depth measurement of the key metabolites and their related compounds. The quantitative results may help understanding the metabolic pathway and the establishment of a diagnostic panel, which enables the diagnostic and treatment applications in the clinic.

## Materials and Methods

### Reagents and Solutions

Mass spectrometry level methanol, acetonitrile, isopropanol, formic acid and ammonium acetate were purchased from Fisher Scientific (Fair Lawn, United States). Mass spectrometry level ammonium bicarbonate and methyl tert-butyl ether (MTBE) were purchased from Sigma-Aldrich (St. Louis, United States). Ultra-pure water (18.2 m Ω cm) was used to prepare using Milli-Q purified water system (Merck KGaA, Darmstadt, Germany). Reference bile acid standards and isotope internal standards were purchased from Avanti Polar Lipids (Alabama, United States), Cayman Chemical (Ann Arbor, United States), Cambridge Isotope Laboratories Inc. (Tewksbury, United States), IsoSciences (Ambler, United States), Sigma-Aldrich (St. Louis, United States) and Toronto Research Chemicals (Toronto, Canada). For more information about standards, please referred to Supplementary Table S1.

### Participants and Criteria

From June 2020 to January 2021, serum samples were collected from osteoporosis patients (OS group, *n* = 32) at the First Affiliated Hospital of Dalian Medical University. The OS group inclusion criteria were based on the 2014 National Osteoporosis Foundation (NOF) clinical guidelines (Cosman et al., 2014). The exclusion criteria included any mental or organic diseases, cancer, metabolic or hereditary bone disease, and hormone use in the past 6 mo. The serum samples of the control group (Con group, *n* = 32) were collected from health individuals at an admission physical examination. The age and sex constituent ratio of the control group matched that of the OS group, and the controls did not have any of the above-mentioned OS group exclusion criteria. All patients signed informed consent forms, and the project was approved by the Ethics Committee of First Affiliated hospital of Dalian Medical University.

### Serum Sample Collection and Pretreatment

Serum samples were collected from OS patients on the first morning in a fasted state. Likewise, all Con samples were collected at the same time point and under the same fasting conditions as the OS samples were. All samples were immediately stored in a −80°C freezer and thawed at 4°C before pre-treatment. First, 150 μl of each sample was transferred to 1 ml 96-DeepWell plates (Thermo Scientific, United States), and then, 600 μl of methanol was added to the sample to precipitate the protein. Next, the mixture was vortexed for 5 min for better mixing and distribution and centrifuged at 5300 RPM for 20 min (4°C). Two replicates of the 200 μl upper layer were transferred to 450 μl 96-well plates (Thermo Scientific, United States); the samples were concentrated and dried by vacuum centrifugation. The polar metabolite extractions in these two plates were redissolved for positive and negative ion detection with untargeted metabolomics analysis. The remaining upper layers of all samples were mixed and similarly distributed at 200 μl per replicate as quality control (QC) samples (Salem et al., 2016).

To extract lipids from serum, 120 μl methanol was added to 20 μl of sample in a 1.5 ml EP tube (Axygen, United States). Next, the mixture was vortexed for 180 s, and 360 μl of methyl tert-butyl ether (MTBE) and 100 μl of ultrapure water were subsequently added to the solution. The mixture was vortexed for 10 min, kept at room temperature for another 10 min, and finally centrifuged at 13,000 × g for 15 min (4°C). 200 μl of lipid extract from the upper layer was transferred to a 1.5 ml EP tube and dried, similar to the protocol for the polar metabolite extractions described above. The lipid extractions were then redissolved for lipidomics analysis. QC samples of lipids were also prepared.

Moreover, a standard curve configuration was essential for metabolite-targeted quantification. Therefore, we precisely weighed the standards and adjusted the concentration to 1.0 mg ml^−1^ as a stock solution. An appropriate volume of each stock solution was diluted step by step to 465 μg L^−1^, 232.5 μg L^−1^, 116.25 μg L^−1^, 58.125 μg L^−1^, 29.0625 μg L^−1^, 14.531 μg L^−1^, 7.266 μg L^−1^, 3.633 μg L^−1^, 1.816 μg L^−1^, 0.908 μg L^−1^, 0.454 μg L^−1^, 0.227 μg L^−1^ and 0.114 μg L^−1^ with the extraction solution. Next, 80 μl of each sample was transferred to 1 ml 96-DeepWell plates, and 320 μl of methanol: acetonitrile (1:1, v:v), which included a 50 ng ml^−1^ bile acid isotope internal standard, was added. After 5 min of vortexing, the mixture was centrifuged at 5300 RPM at 4°C for 20 min. The 260 μl upper layer was transferred to 450 μl 96-well plates and dried as described above. Afterwards, the extraction was redissolved for bile acid-targeted metabolomics analysis. The mixture of QC samples was also distributed at 260 μl per replicate and dried (Choucair et al., 2020).

### Untargeted Metabolomic and Lipidomic Analysis

The UHPLC-HRMS system, which was used for untargeted metabolomics analysis, was composed of an Ultimate 3000 ultra-high performance liquid chromatograph and Q Exactive Quadrupole-Orbitrap High-Resolution Mass Spectrometer (Thermo Scientific, United States).

The polar metabolite extracts were separated by reversed-phase chromatography for positive and negative ion detection. Metabolites were separated by using an Excel 2 C18-PFP column (3.0 μm, 2.1 × 100 mm; ACE Co., United Kingdom) for positive detection and eluted with 0.1% formate/water as mobile phase A and acetonitrile as mobile phase B. The linear gradient ramped from 2% mobile phase B to 98% in 10 min. For the negative detection mode, the mobile phases consisted of water (phase A) and acetonitrile/methanol (phase B), both of which contained ammonium bicarbonate buffer salt, and were employed to elute metabolites separated on an Acquity HSS C18 column (1.8 μm, 2.1 × 100 mm; Waters Co., United States). The mobile phase gradient was as follows: 0 min 2% phase B ramped to 100% in 10 min, and another 5 min was used for column washing and equilibration. The flow rate, injection volume and column temperature of both the positive and negative modes were set at the same conditions: 0.4 ml min^−1^, 5 μl and 50°C.

The chromatographic separation for lipidomic was carried out in positive ionization detection mode. An Accucore C30 core-shell column (2.6 μm, 2.1 × 100 mm; Thermo Scientific, United States) was utilized for lipid molecule separation at 50°C, and the lipids were eluted with 60% acetonitrile in water (phase A) and 10% acetonitrile in isopropanol (phase B), both of which contained 10 mM ammonium formate and 0.1% formate. The separation gradient was optimized as follows: initial 10% B ramping to 50% in 5 min and further increasing to 100% in 23 min. The other 7 min were used for column washing and equilibration using a 0.3 ml min^−1^ flow rate.

For polar metabolite detection, the Quadrupole-Orbitrap mass spectrometer was operated under identical ionization parameters with a heated electrospray ionization source except ionization voltage: sheath gas, 45 arb; aux gas, 10 arb; heater temperature, 355°C; capillary temperature, 320°C and S-Lens RF level, 55%. The metabolomic extracts were profiled in full scan mode under 70,000 FWHM resolution with AGC 1 × 10^6^ and 200 ms max injection time. Data were acquired using a scan range of 70–1,000 m z^−1^. The lipid molecules were ionized using the same parameters mentioned above. At a 70,000 full width half maximum (FWHM) full scan resolution, the settings differing from those of the polar metabolite analysis included the 300–2000 m z^−1^ scan range and AGC target 3 × 10^6^.

### Targeted Metabolomics Analysis

A total of 66 bile acids (Supplementary Table S2) were scanned and quantified on a Waters Acquity UPLC (Waters Corp., Milford, United States) coupled with a Sciex 5500+ triple quadrupole (QQQ) mass spectrometer (AB Sciex, Singapore). The bile acids were chromatographically resolved on an C18-PFP column (3 μm, 2.1 × 50 mm; ACE, United Kingdom) after 2.5 μl aliquots of bile acid extract was injected. Water containing 2 mM ammonium acetate was used as phase A, and acetonitrile was used as phase B. The chromatographic gradient ramped from 17% phase B to 30% in 10 min, ramped to 55% in 3 min, rapidly climbed to 95% in 1 min and remained for 3 min; another 5 min was used for column washing and equilibration. The flow rate was set at 0.4 ml min^−1^. The metabolites were ionized by a TurboV heated electrospray ionization source and then detected by scheduled multiple reaction monitoring mode. The main parameters were optimized as follows: negative ion spray voltage was −4.5 kV, curtain gas pressure was 35 psi, ion gas 1 and 2 pressure were 50 psi, and heater temperature was 550°C.

### Date Processing

According to the recommendation of the Metabolomics Standardization Initiative (MSI) (Sumner et al., 2007), first-level annotation required chromatographic retention time, primary mass spectrometry and secondary mass spectrometry information, which was consistent with the standards. At the second level, the polar metabolites were structurally annotated by searching against local databases, mzCloud library (Thermo Scientific, United States), Kyoto Encyclopedia of Genes and Genomes (KEGG) and the Human Metabolome Database (HMDB). On the other hand, untargeted lipid data were processed with LipidSearch (Thermo Scientific, United States) software, including peak picking and lipid identification. For metabolite identification or structural annotation, accuracy of the mass of a precursor within ±10 ppm was a prerequisite. The AUC values were extracted as relative quantification information of polar metabolites and lipids with TraceFinder software (Thermo Scientific, United States). Regarding targeted bile acid detection, internal calibration was conducted with Analyst software and OS-MQ software (AB SCIEX, Singapore) for quantitative analysis of bile acids.

### Statistical Analysis

We used R package “pwr” for classical Power Analysis. Next, metabolites with missing value percentages above 50% were excluded, and then the K-nearest algorithm (KNN sample-wise) was employed to impute the missing values. For the purpose of guaranteed uniqueness of metabolites and lipids, molecules detected by multiple methods were retained only once. The normalization of untargeted metabolomics data consisted of three steps: sample calibration, data transformation and data scaling. Firstly, Sample calibration was used to correct sample reproducibility due to batch effects or systematic errors in detection. Secondly, we performed Log transformation on untargeted metabolomics data, which was often used to convert data into normal distribution. Finally, UV scaling was used to pre-process orthogonal projections to latent structures discriminant analysis (OPLS-DA) data. For the bile acid targeted analysis, the mass concentration of serum extraction was transformed to the molar concentration of the original sample based on the molecular weight and dilution factor. Multivariate analysis, such as principal component analysis (PCA) and OPLS-DA, was conducted with SIMCA-P software (Umetrics, Sweden). Univariate analysis including independent samples Student’s t-test *p*-value, Benjamini-Hochberg false discovery rate q-value (*p*-value < 0.05, q-value < 0.2) (Oshansky et al., 2014; Park et al., 2019; Shi et al., 2020, 16) and heatmap drawing was performed on the MetaboAnalyst website (http://www.metaboanalyst.ca) (Xia and Wishart, 2010b, 2010a; Chong et al., 2018). We applied the WGCNA package in the R environment Version 4.0.3 (R Core Team, 2020) to construct co-expression modules of highly correlated metabolites (Langfelder and Horvath, 2008). Moreover, receiver operating characteristic (ROC) curves and box plots were generated with GraphPad Prism 8.0 (GraphPad Software Inc., United States). Binary logistic regression and biomarker model establishment were based on SPSS Statistics 26.0 software (IBM, United States). Cytoscape 3.8.0 (https://cytoscape.org/, Cytoscape Consortium, United States) was used for biological network construction and visualization (Shannon et al., 2003).

## Results

### Study Design and Clinical Characteristics

**FIGURE 1:**
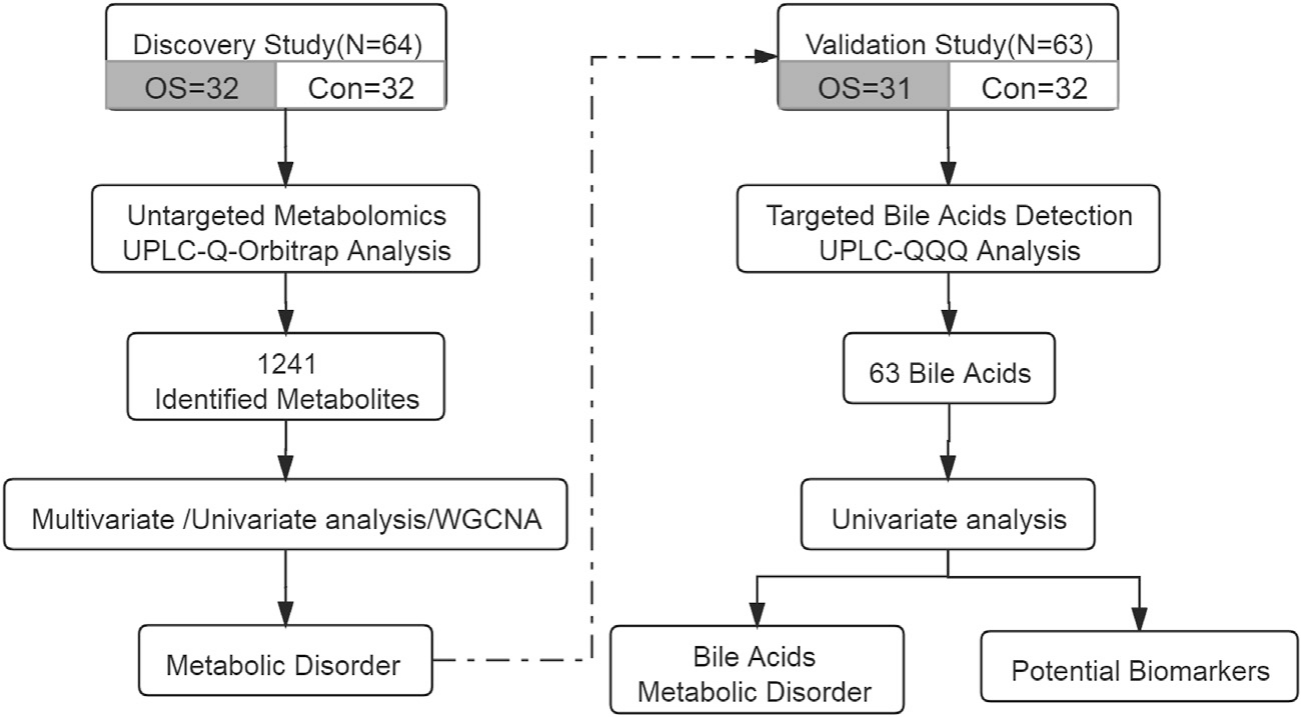
The design and workflow of this study.

Serum samples from 32 patients with osteoporosis and 32 healthy individuals were collected. The annotated serum metabolites were compared and clustered by multivariate analysis, univariate analysis and WGCNA. To clarify the results, targeted analysis of bile acids was performed using another aliquot of the serum samples from the same two groups. Diagnostic model was established using the quantitative BAs’ data. The workflow of this study was summarized in Figure 1.

No significant differences were found in the clinical characteristics including sex, age, glucose, creatinine, and etc. between the matched groups of Controls and OS. Detailed clinical information is listed in Supplementary Table S3.

Untargeted metabolomics was employed to describe the characteristics of serum metabolism among the participants. A total of 1,241 metabolites (1,083 metabolites remaining after data screening and cleaning) were identified. In addition, 366 polar metabolites accounted for 33.8% of the total, and 717 lipids accounted for 66.2%. Among them, 266 triacylglycerols (TGs) accounted for the largest proportion (24.6%). The total ion chromatogram (TIC) displayed the panoramic view of non-targeted metabolomics. The extracted ion chromatogram (XIC) provided a visual presentation of targeted bile acid detection (Supplementary Figure S1). The coefficient of variation (CV) distribution of QC, which indicated the reproducibility of the detection method, is shown in Supplementary Figures S2,S3.

### Metabolic Profiling of Osteoporosis

**FIGURE 2:**
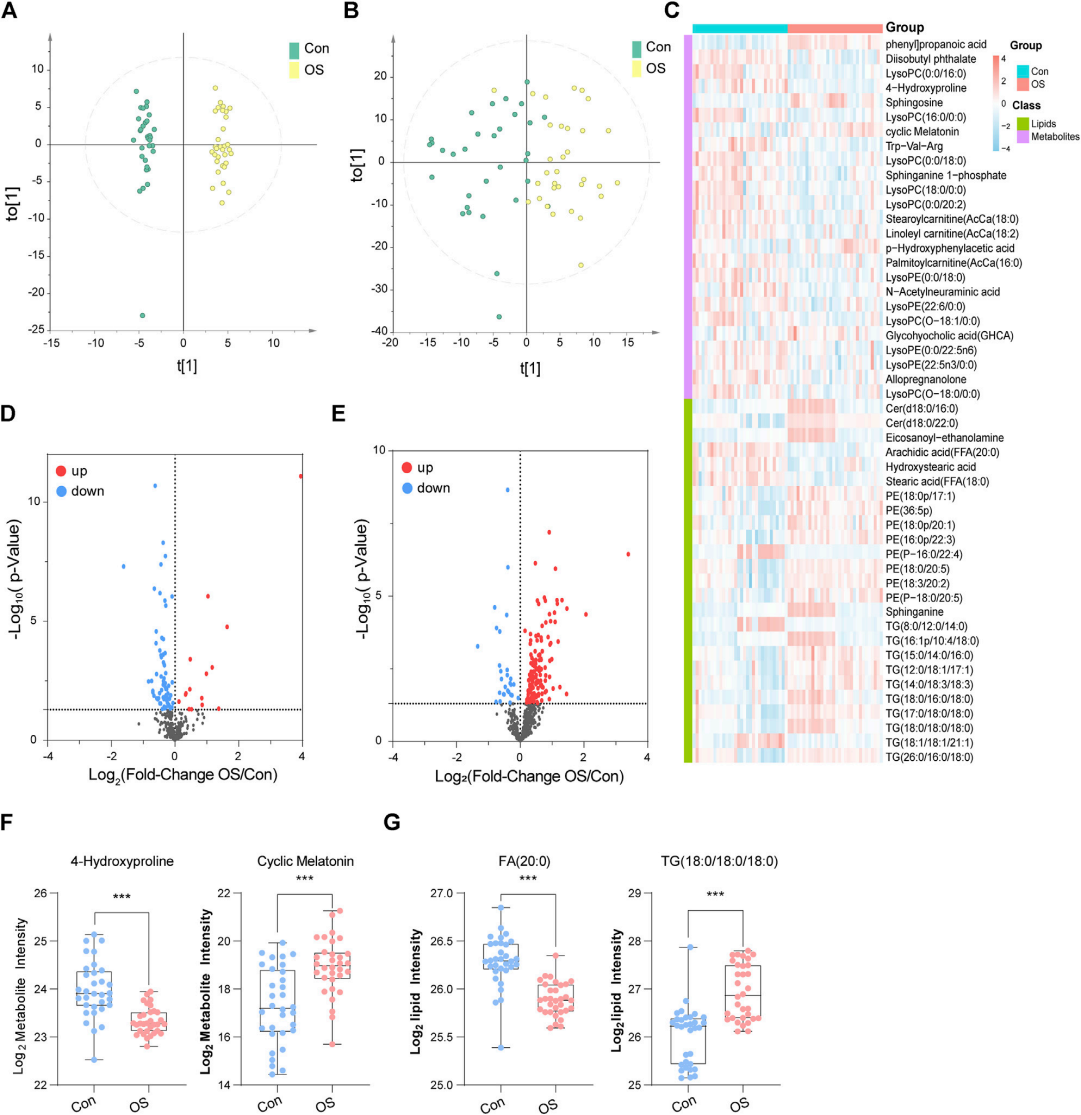
OPLS-DA score plots of metabolites **(A)** and lipids **(B)**. In the heatmap, blue indicates lower relative intensity, and red indicates higher relative intensity **(C)**. The red dots of the metabolites **(D)** and lipids **(E)** in the volcano plots indicated an increase in the OS group, and the blue dots indicate a decrease. 4-Hydroxyproline and cyclic melatonin were representative metabolites **(F)**. FA (20:0) and TG (18:0/18:0/18:0) were representative lipids **(G)**.

To illustrate the metabolic alterations between the two groups, OPLS-DA of polar metabolites (Figure 2A) and lipids (Figure 2B) were used. An overall separation can be observed between the two groups, in both platforms. Volcano plots of polar metabolites (Figure 2D) and lipids (Figure 2E) show the differences and average intensity change ratio between the two groups. Most of the polar metabolites decreased, while the lipids increased in the OS group. The relative contents of these metabolites could be visualized by a heat map (Figure 2C). The differential metabolites identified mainly included amino acids (AAs), fatty acids (FAs), glycerophosphocholines (PCs), glycerophosphoethanolamine (PE), TGs and BAs. Notably, the serum contents of lysophosphocholines (LPC) in the OS group was significantly lower than that in the Con group, which was in contrast to the trends of other lipids, such as PE and TG. In Figures 2F,G, 4-Hydroxyproline and FA (20:0) levels decreased significantly in the OS group, while cyclic Melatonin and TG (18:0/18:0/18:0) levels increased obviously in the OS group (Supplementary Tables S4,S5).

### Construction of Co-expression Modules by WGCNA

**FIGURE 3:**
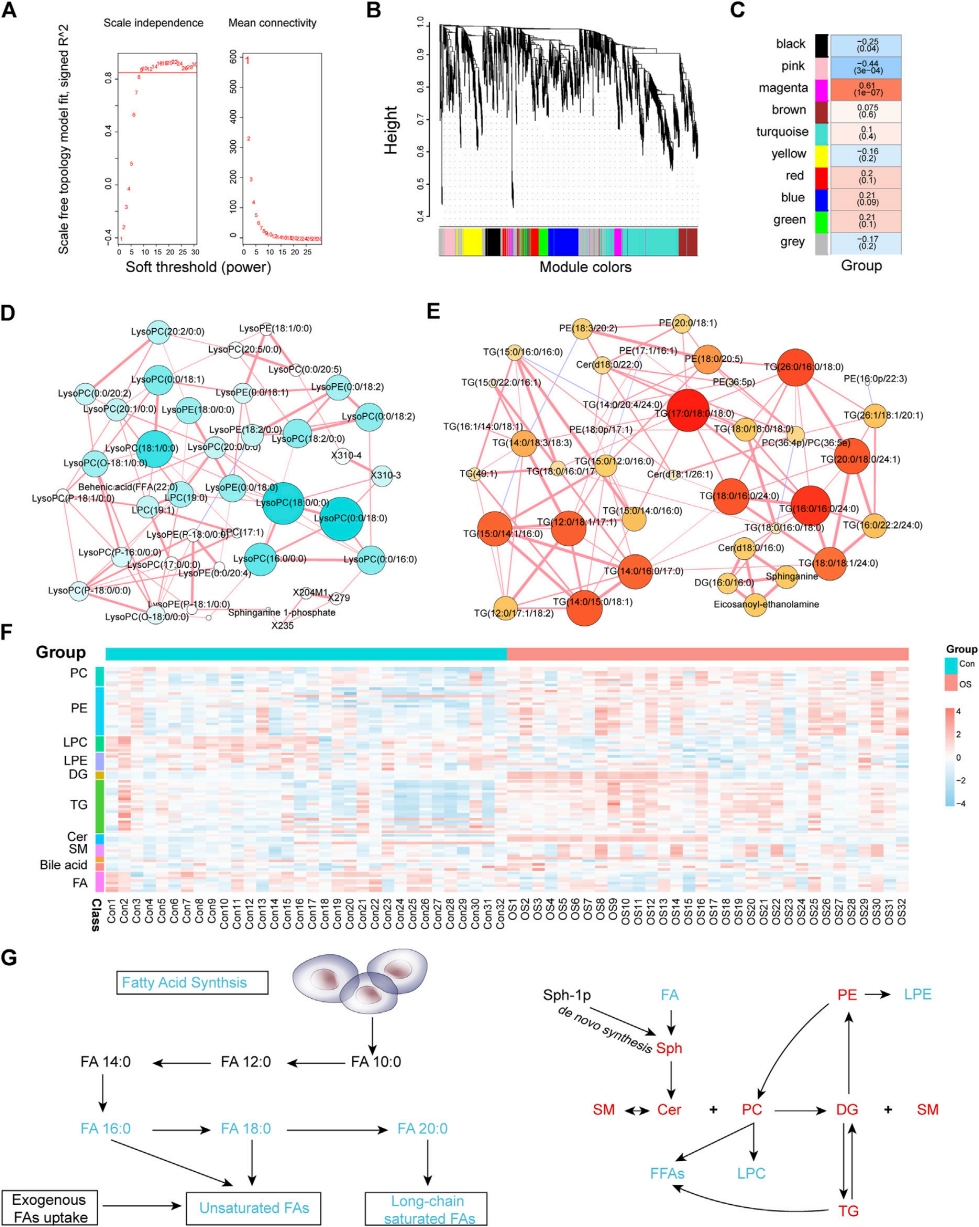
Soft threshold selection was based on scale-free topology R2 and mean connectivity **(A)**. A module cluster tree was used to visualize the distribution of metabolites in each module **(B)**. The correlation coefficients and *p*-values between modules and osteoporosis **(C)**. The MMI networks of the pink **(D)** and magenta **(E)** modules. The correlation of MMI was based on debiased sparse partial correlation (DSPC). The heatmap of osteoporosis-related metabolites and lipids that were differentially expressed between the OS and Con groups **(F)**. Schematic plot of FA synthesis metabolism and the mutual transformation between subclasses of lipids **(G)**. Black text represents undetected metabolites, red text represents significantly enriched metabolites, and green text represents significantly depleted metabolites when the OS group is compared with the Con group.

To find out the interaction of the differential metabolites, WGCNA, an innovative analysis method, was used to construct a metabolite interaction network considering weighted factors. According to the relative intensity data of 1,083 metabolites, the correlation between the metabolic co-expression module and the clinical phenotype of osteoporosis was analysed by the WGCNA software package. First, the hierarchical clustering method was used to check the outliers, and no outliers were found. The soft threshold was 9 (scale-free topology R2 = 0.882, slope = −1.29, mean connectivity = 20.5); subsequently, the merged cut height was set to 0.2 with a minimum module size of 30. A total of 10 modules were obtained, among which, the grey module was a group of metabolites that could not be included in the co-expression network construction. This module should be reduced as much as possible for the robustness of the model. Pearson correlation analysis was used to evaluate the correlation between modules (Supplementary Table S6). The results suggested that there was a significant positive correlation among the green, red and blue modules and that there were no significant negative correlations among modules. The differences between the osteoporosis patients and healthy controls were the clinical features those are concerned about. From Figure 3C, we found that the pink (Supplementary Table S7) and magenta (Supplementary Table S8) modules were significantly related to the occurrence of osteoporosis. The metabolites in pink were expressed at low levels in the OS group, while the change trend of the metabolites in the magenta module was the opposite.

### Metabolite-Metabolite Interaction (MMI) Network Construction

The differences between the OS group and Con group were compared in the two modules. The pink module contained 43 metabolites, of which 25 metabolites were significantly downregulated and none upregulated (Supplementary Figure S4G). In addition, levels of all 45 metabolites in the magenta module were significantly elevated (Supplementary Figure S4I). Furthermore, metabolites with the greatest fold change (FC) ratio between the two groups in each module were selected as representatives. LPC (18:0/0:0) and LPC (16:0/0:0) in the pink module decreased significantly in the OS group. In contrast, TG (17:0/18:0/18:0) and TG (16:0/16:0/24:0) were significantly higher in the OS group than in the Con group. To identify the relationship between the key metabolites in each module, an MMI network was constructed based on the internal connectivity of the metabolites. Surprisingly, the MMI of the pink module (Figure 3D; Supplementary Table S9) indicated that among the various metabolites downregulated in the OS group, the highly weighted metabolites were mainly LPC. Moreover, the MMI of the magenta module suggested that the upregulated metabolites in the OS group were mainly TG and PE (Figure 3E; Supplementary Table S10).

### Disorders of Lipids Pathways

Patients with osteoporosis had significant abnormal lipid metabolism (Supplementary Table S11). As shown in Figures 3F,G, levels of PC, PE, diacylglycerols (DG), TG, ceramides (Cer) and sphingomyelins (SM) in the serum of osteoporosis patients were dramatically increased. The relative serum concentrations of LPC, lysophosphatidylethanolamine (LPE), Acetylcarnitine and FA, especially saturated FA, decreased in patients with osteoporosis. In addition, most of the FA chains in TG and PE were long-chain saturated FAs. The changes in different BAs also varied between the two groups. These characteristics indicated that dysregulation of lipid metabolism may contribute to the occurrence of osteoporosis.

### Dysregulation of BAs

**FIGURE 4:**
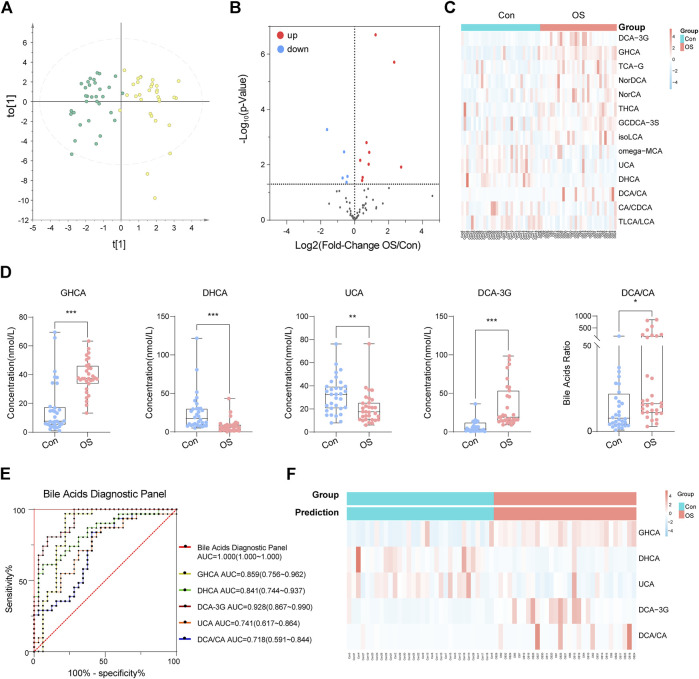
OPLS-DA plot of absolute quantitation of BAs **(A)**. Volcano plot of absolute quantitation of BAs **(B)**. Heatmap of selected BAs that were significantly changed in the volcano plot **(C)**. After analysis by binary logic regression, five BA biomarkers were visualized in the form of box plots **(D)**. The ROC curve of the above five biomarkers and the diagnostic panel **(E)**. The prediction accuracy of the diagnostic panel is shown by a heatmap **(F)**.

To validate whether abnormal bile acid metabolism is involved in the occurrence of osteoporosis, a targeted method was carried out on the same batch of serum samples (Supplementary Table S12). 49 bile acids were detected from the samples. Similarly, the OPLS-DA score plot showed a significant separation between the OS group and the Con group (Figure 4A). The volcano plot indicated that five BAs (or ratios) decreased and that 11 increased in osteoporosis (Figure 4B). Furthermore, the concentrations of 16 BAs (or ratios) in each sample are shown in a heatmap (Figure 4C).

A diagnostic panel was established based on differentially expressed BAs using binary logistic regression. After variable screening, the box plot of five potential BAs, glycohyocholic acid (GHCA), dehydrocholic acid (DHCA), deoxycholic acid 3-glucuronide (DCA-3G), ursocholic acid (UCA), and deoxycholic acid/cholic acid (DCA/CA), showed that GHCA, DCA-3G, and DCA/CA levels in osteoporosis patients were significantly higher than those in healthy controls. In addition, DHCA and UCA, which were classified as hyocholic acid species (HCAs), were higher in healthy controls (Figure 4D). This diagnostic panel for osteoporosis was concluded as follows: Logit[P]=24.063×GHCA-53.524×DHCA-21.971×UCA+54.302×DCA-3G+0.615×DCA/CA-123.056 In the equation, P is the predicted probability of osteoporosis, and each BA represents its serum concentration (nmol L^−1^). The AUC values of the five bile acid biomarkers were as follows: GHCA, AUC = 0.859 (0.7756–0.962); DHCA, AUC = 0.841 (0.744–0.937); DCA-3G, AUC = 0.928 (0.867–0.990); UCA, AUC = 0.741 (0.617–0.864); and DCA/CA, AUC = 0.718 (0.591–0.844). Noticeably, performance of the diagnostic panel in the diagnosis of osteoporosis was superior to that of each bile acid biomarker alone (Figure 4E). Moreover, the prediction accuracy of this diagnostic panel was 100% (Figure 4F). The results highlighted the diagnostic potential of bile acids.

## Discussion

In the present study, we characterized the differences in metabolite and lipid profiles between osteoporosis patients and healthy volunteers using LC-MS metabolomics. WGCNA was utilized to identify metabolites that are closely related to osteoporosis onset. Lipid metabolism disorders, mainly abnormalities in fatty acid metabolism, sphingolipid metabolism and BA metabolism, were involved in the initiation of osteoporosis. BAs are involved in lipid digestion and are also important signalling molecules in lipid metabolism. The difference in BAs between osteoporosis patients and healthy volunteers was significant, especially in HCAs. Moreover, the AUC of the 5-metabolite panel provides a promising diagnostic potential. These results demonstrated the role of BAs in osteoporosis.

LPC and LPE are components molecular of membrane and take part in signal transduction (Bai et al., 2014; Rindlisbacher et al., 2018). These compounds are converted from PC and PE by phospholipase A2 (PLA2), a calcium-dependent protein (Hirabayashi et al., 2017). The levels of lyso-lipids were decreased in patients with osteoporosis, while PC and PE levels were increased. The results implied that PLA2 enzyme activity is decreased due to disorders of calcium and phosphorus metabolism, leading to a decrease in LPC, LPE and free fatty acid (FFA) levels. Studies have shown that LPC has pro-inflammatory activity and promotes osteoblast apoptosis (Brys et al., 2019). The accumulation of LPC in bone tissue may lead to the decrease of serum LPC.

Cholesterol is the precursor of vitamin D, bile acids, and steroid hormones, all of which are important regulators of bone metabolism (Hoppel, 2003; Hernandez et al., 2019). BAs regulate the homeostasis of cholesterol, glucose and fat-soluble vitamins, and play a crucial role in maintaining mineral homeostasis (Ma and Patti, 2014; Ruiz-Gaspà et al., 2020). DCA and TCA differed notably in bone tissues of old mice and young mice models of osteoporosis in the literature (Nam et al., 2018). In our results, BAs changed significantly among osteoporosis patients. However, no significant differences in serum CA, chenodeoxycholic acid (CDCA), DCA and lithocholic acid (LCA). Nevertheless, their derivatives, such as DCA-3G, 23-nordesoxycholic acid (Nor-DCA) and isolithocholic acid (iso-LCA), were significantly different between the two groups. Further exploration of the functions of these BAs is needed.

Interestingly, a significant deregulation of HCAs was found in this study. HCAs are a group of 6a-hydroxylated bile acids that account for a minimal proportion of the total BAs in humans and mice but constitute nearly 80% of BAs in pigs (Spinelli et al., 2016). A recent study reported that HCAs were involved in maintaining glucose homeostasis. HCA promoted glucagon-like peptide-1 (GLP-1) production in enteroendocrine cells by simultaneously activating the membrane G protein-coupled receptor TGR5 and inhibiting farnesoid X receptor (FXR) in a dose-dependent manner, which enhanced insulin secretion and eventually reverted to normoglycaemia (Zheng et al., 2021). Epidemiological investigations found that patients with diabetes have a higher risk of osteoporosis. Succinate enhanced osteoclasts by activating succinic acid receptors in diabetes-associated osteoporosis (Guo et al., 2017). However, in this study, GHCA and taurohyocholic acid (THCA) levels were significantly increased, but there was no significant difference in blood glucose between the two groups. Therefore, the correlations between HCAs and osteoporosis are independent from the occurrence of diabetes.

**FIGURE 5:**
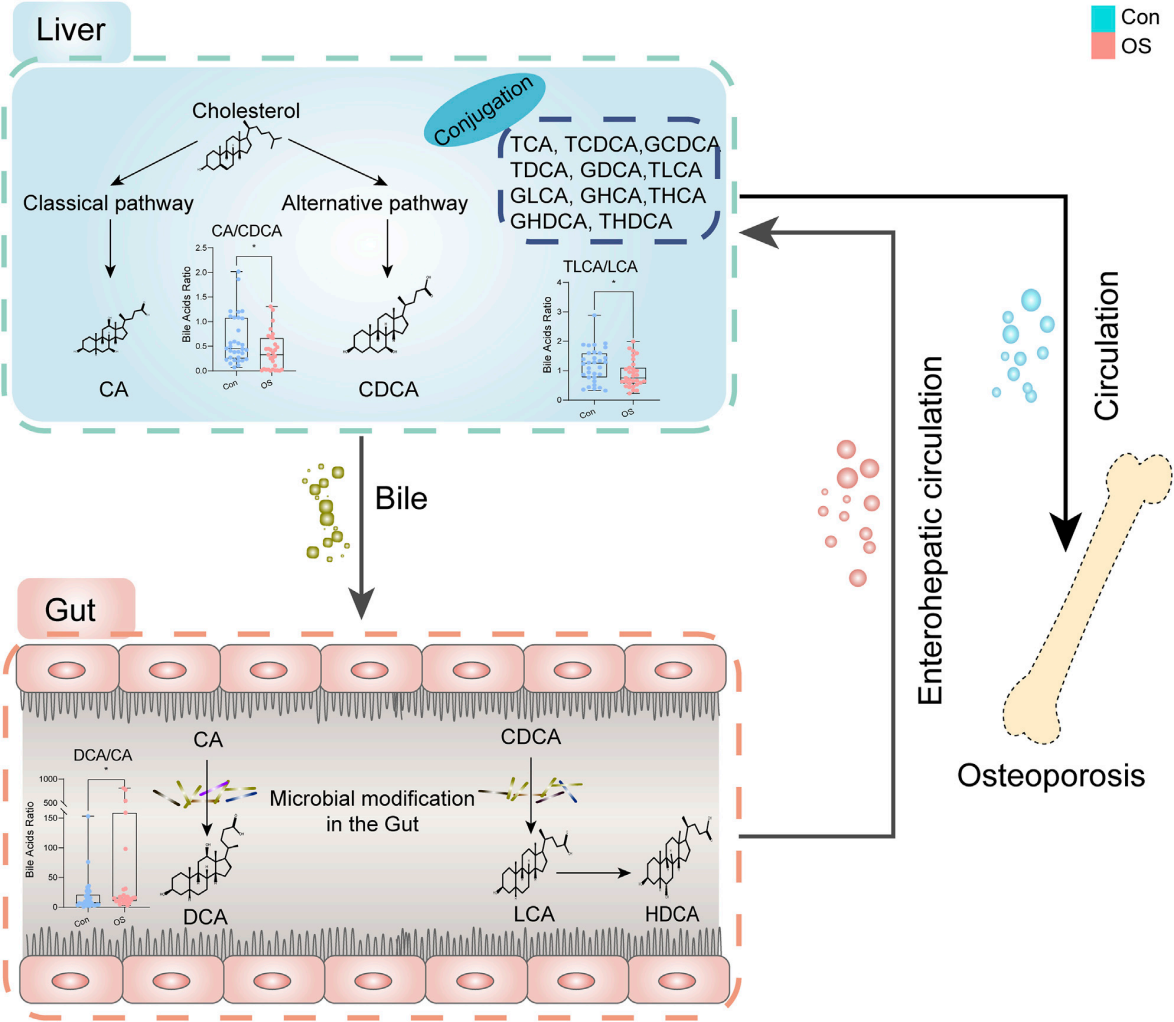
Dysregulation of BA metabolism in osteoporosis.

The molecular ratio of the upstream and downstream of the metabolic pathway is usually used to reflect the catalytic enzymatic activity (MahmoudianDehkordi et al., 2019; Nho et al., 2019). To investigate the mechanisms contributing to BA alterations in osteoporosis, the ratios of three types of bile acids were compared. The results revealed that bile acid metabolism was converted from the classical pathway to the alternative pathway (CA: CDCA). Since the gut microbiota is believed to be closely connected to osteoporosis, the dysregulation of the gut flora may alter BA levels consequently (Jones et al., 2014; Devlin and Fischbach, 2015; Wahlström et al., 2016). A significant change in secondary BAs was found according to the ratio (DCA:CA). CA is affected by bacterial 7A-dehydroxylase in the gut to produce DCA, which has cytotoxic effects and can result in the destruction of the mitochondrial membrane (Schulz et al., 2013). There was a change in the progression of taurine conjugation of secondary BAs in the liver (taurolithocholic acid (TLCA): LCA). These results indicated that gut microbiota and related BA metabolism may act as an important role in the occurrent osteoporosis (Figure 5).

Although our study provided original insights into the pathogenesis of osteoporosis, there were still some limitations. First, the sample size was relatively small, and therefore, we could not stratify the metabolites associated with disease progression according to severity. Second, although the diagnostic model had good diagnostic performance, it still needs to be validated in a larger cohort. Finally, this study was a retrospective study, and the causal relationship between differential metabolites and osteoporosis requires further investigation.

## Conclusion

Our integrated metabolomic strategy was demonstrated to be practical for the screen of novel biomarkers, which highlights the lipids and bile acids metabolism disorders in patients with osteoporosis. Bile acids change from the classical pathway to the alternative pathway, and HCAs are involved in the occurrence and development of osteoporosis. The deregulation of lipids and the BAs provides a potential basis for the diagnosis and treatment of osteoporosis. Our study confirmed the importance of the combination of untargeted and targeted metabolomic method especially for the trnslational research in the clinic.

## Data Availability

The original contributions presented in the study are included in the article/Supplementary Material, further inquiries can be directed to the corresponding author/s.
