# Supplementary figures and images for: An Integrated Metabolomic Study of Osteoporosis: Discovery and Quantification of Hyocholic Acids as Candidate Markers
Source: Front Pharmacol. 2021 Aug 6;12:725341. doi: 10.3389/fphar.2021.725341 (PMC8378234; doi:10.3389/fphar.2021.725341)

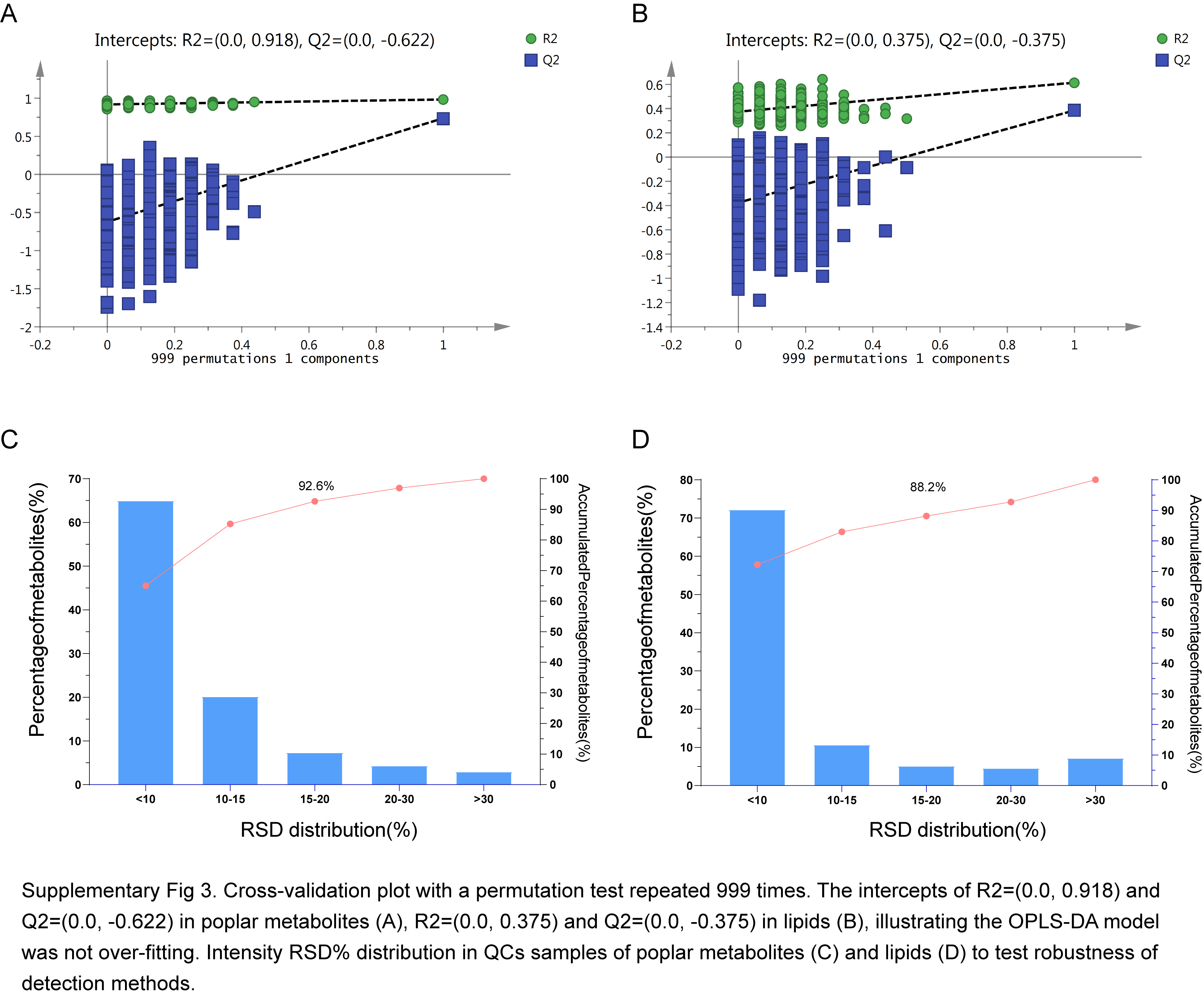

Supplement: Supplementary file 1 [file Image3.TIF]

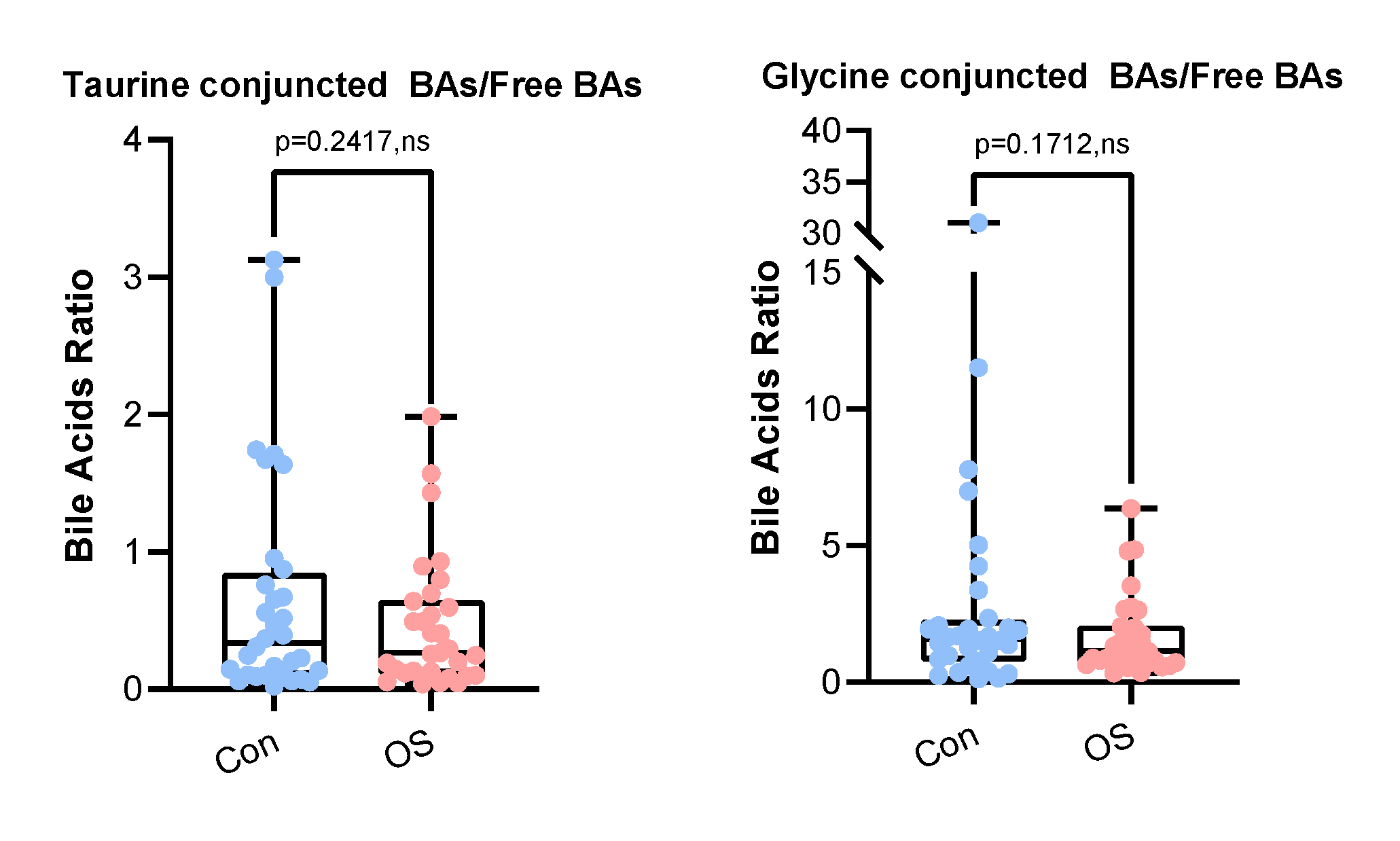

Supplement: Supplementary file 2 [file Image5.TIFF]

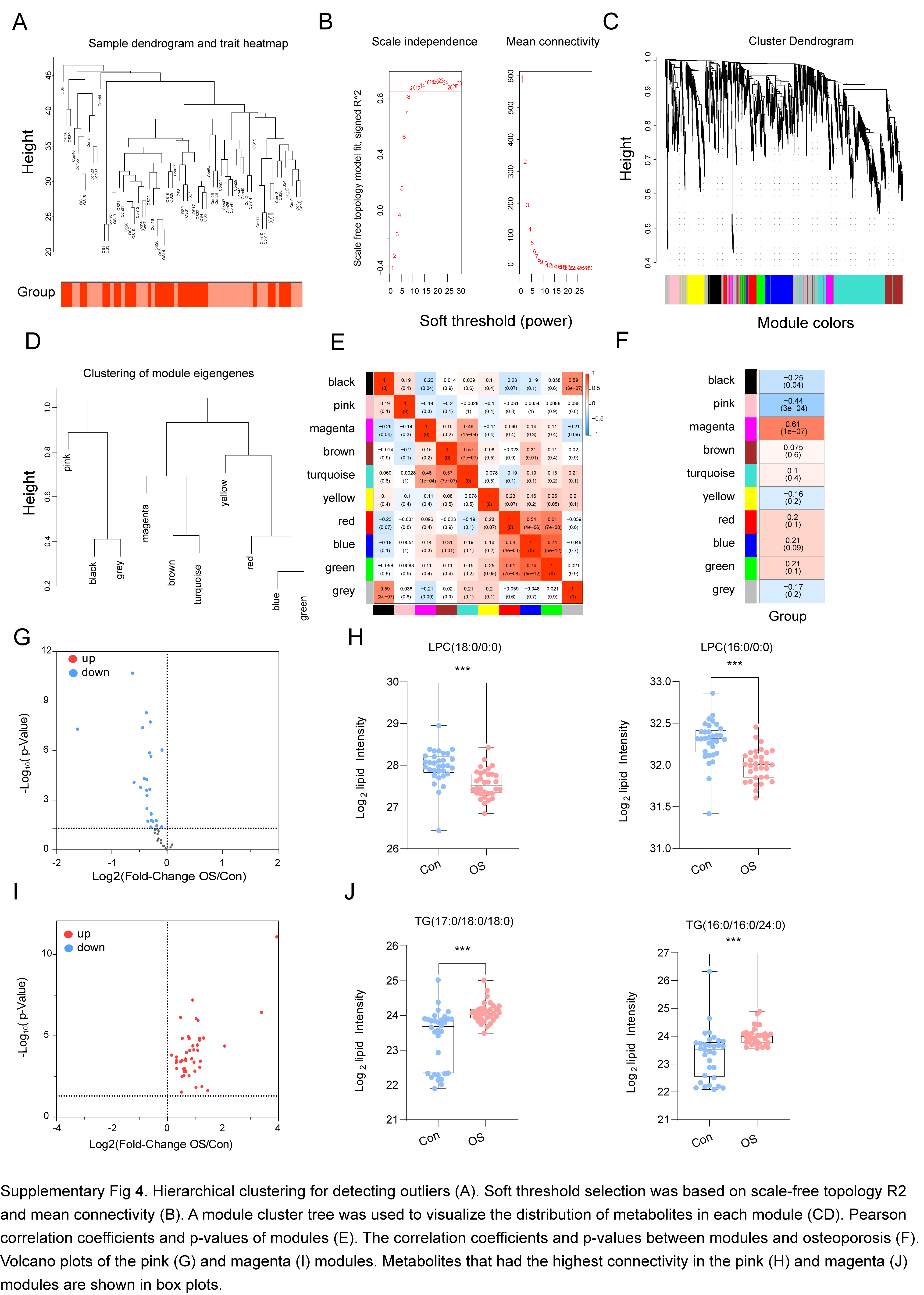

Supplement: Supplementary file 3 [file Image4.TIF]

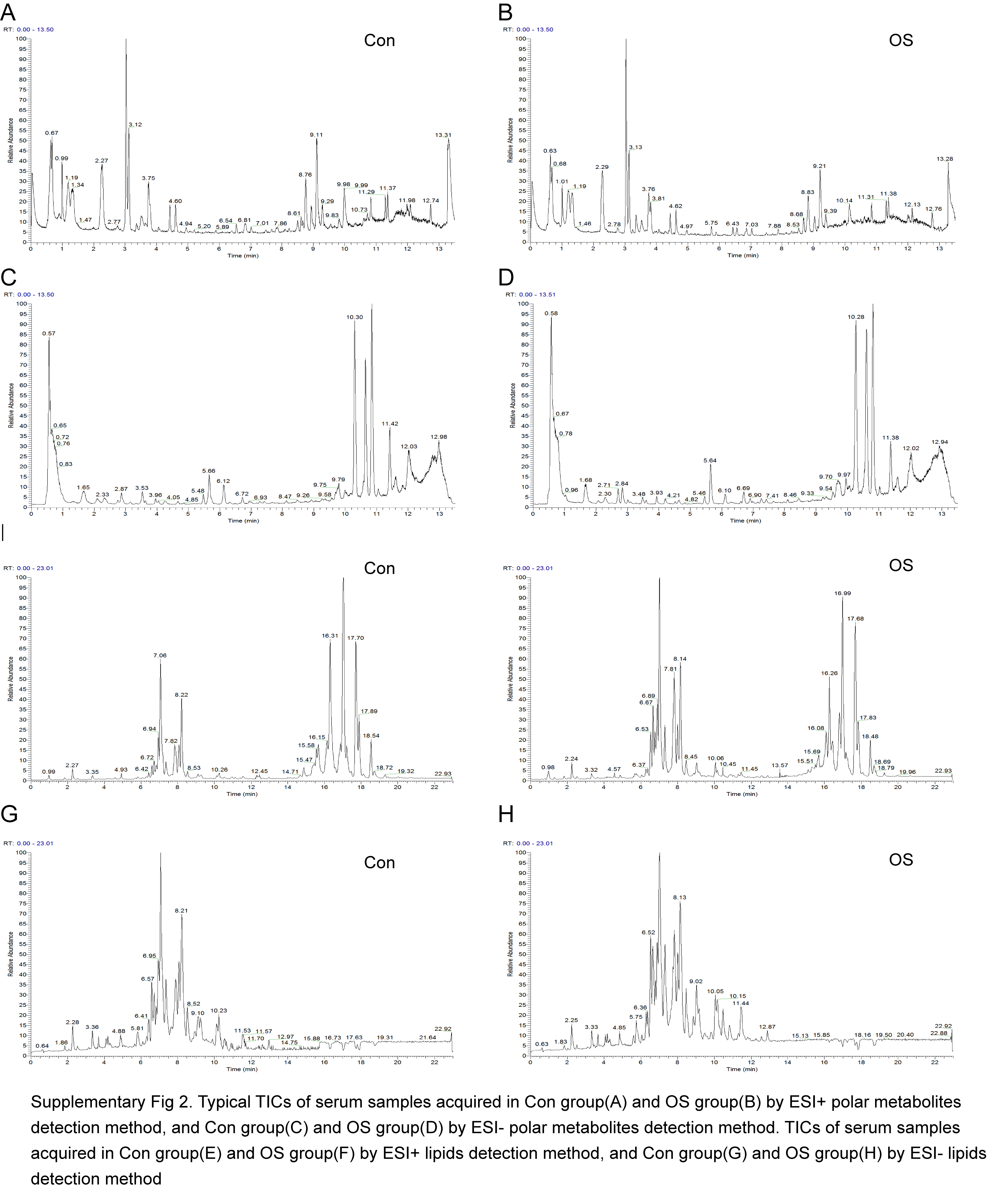

Supplement: Supplementary file 4 [file Image2.TIF]

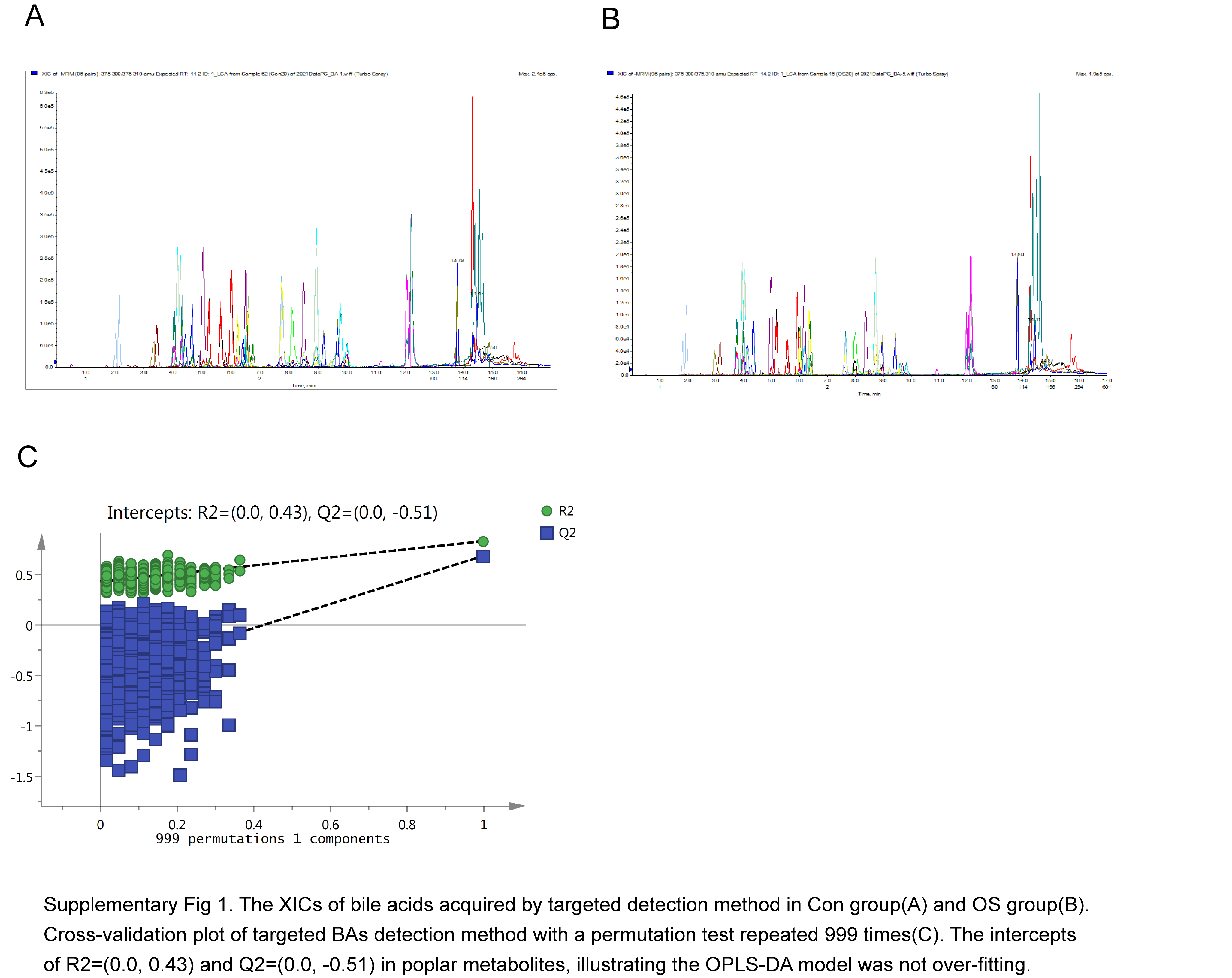

Supplement: Supplementary file 5 [file Image1.TIF]
